# Supplementary material for: A novel fungal metal-dependent α-l-arabinofuranosidase of family 54 glycoside hydrolase shows expanded substrate specificity
Source: Sci Rep. 2021 May 26;11:10961. doi: 10.1038/s41598-021-90490-2 (PMC8155123; doi:10.1038/s41598-021-90490-2)
Supplement: Supplementary file 1 — Supplementary Information 1. [file 41598_2021_90490_MOESM1_ESM.docx]

**A novel fungal metal-dependent α-L-arabinofuranosidase of family 54 glycoside hydrolase shows expanded substrate specificity**

### Maria Lorenza Leal Motta^1^, Jaire Alves Ferreira Filho¹, Ricardo Rodrigues de Melo^2^ , Leticia Maria Zanphorlin^2^, Clelton Aparecido dos Santos^2^, Anete Pereira Souza^1*^

^1^Center for Molecular Biology and Genetic Engineering (CBMEG), University of Campinas (UNICAMP), Campinas, SP, Brazil

^2^National Biorenovables Laboratory, National Research Center for Energy and Materials, Campinas, SP, Brazil

*Corresponding author

**SUPPLEMENTARY INFORMATION**

**Supplementary Figures S1 and S2.**


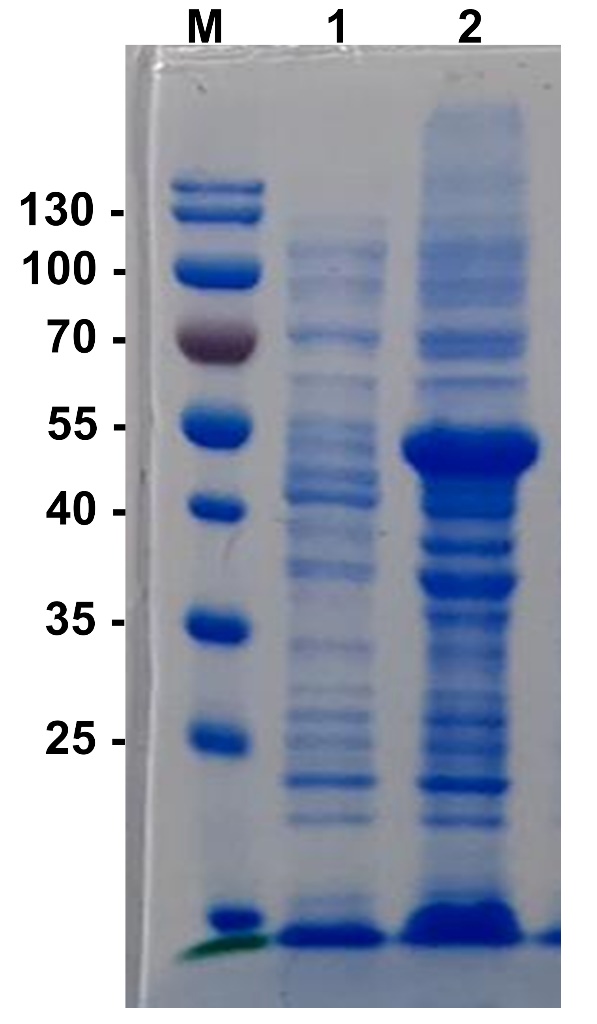


**Supplementary Fig. S1. SDS-PAGE analysis of the soluble and insoluble fractions of ThABF purification**. M indicates the molecular weight marker, 1 the soluble fraction and 2 the insoluble fraction. The gel was stained with a Coomassie blue solution for 30 minutes.


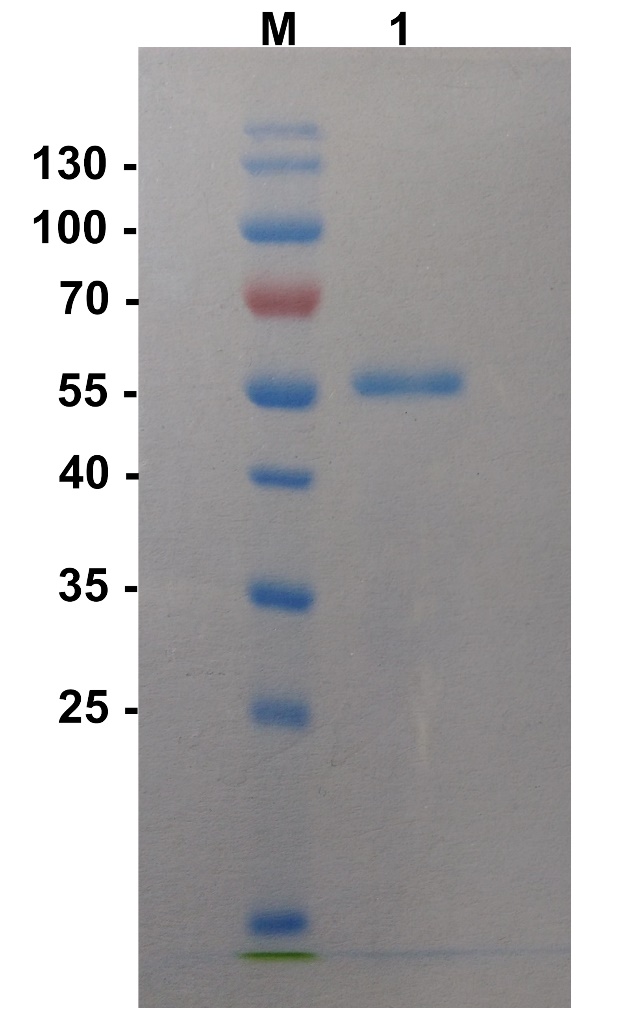


**Fig. Supplementary S2. SDS-PAGE analysis of ThABF purified from inclusion bodies.** M indicates the molecular weight marker and 1 is ThABF at approximately 53.44 kDa. The gel was stained with a Coomassie blue solution for 30 minutes.
